# Investigating the reliability of metapodials as taxonomic Indicators for Beringian horses

**DOI:** 10.1007/s10914-022-09626-4

**Published:** 2022-09-21

**Authors:** Zoe Landry, Mathew J. Roloson, Danielle Fraser

**Affiliations:** 1 Department of Earth Sciences, University of Ottawa, 25 Templeton Street, Ottawa, ON K1N 6N5 Canada; 2 Beaty Centre for Species Discovery, Canadian Museum of Nature, PO Box 3443, Station D., Ottawa, ON K1P 6P4 Canada; 3 Department of Earth Sciences, Carleton University, 1125 Colonel By Drive, Ottawa, ON K1S 5B6 Canada; 4 Department of Biology, Carleton University, 1125 Colonel By Drive, Ottawa, ON K1S 5B6 Canada; 5 Paleobiology Smithsonian National Museum of Natural History, DC 20560 Washington, USA

**Keywords:** Palaeontology, Beringia, Horses, Equus, Metapodials, Taxonomy

## Abstract

The metapodials of extinct horses have long been regarded as one of the most useful skeletal elements to determine taxonomic identity. However, recent research on both extant and extinct horses has revealed the possibility for plasticity in metapodial morphology, leading to notable variability within taxa. This calls into question the reliability of metapodials in species identification, particularly for species identified from fragmentary remains. Here, we use ten measurements of metapodials from 203 specimens of four Pleistocene horse species from eastern Beringia to test whether there are significant differences in metapodial morphology that support the presence of multiple species. We then reconstruct the body masses for every specimen to assess the range in body size within each species and determine whether species differ significantly from one another in mean body mass. We find that that taxonomic groups are based largely on the overall size of the metapodial, and that all metapodial measurements are highly autocorrelated. We also find that mean body mass differs significantly among most, but not all, species. We suggest that metapodial measurements are unreliable taxonomic indicators for Beringian horses given evidence for plasticity in metapodial morphology and their clear reflection of differences in body mass. We recommend future studies use more reliable indicators of taxonomy to identify Beringian horse species, particularly from localities from which fossils of several species have been recovered.

**Supplementary Information:**

The online version contains supplementary material available at 10.1007/s10914-022-09626-4.

## Introduction

Morphological characteristics of organisms are the combined product of their evolutionary history as well as developmental and functional constraints (Wagner and Altenberg 1996; Gould 2002; Hallgrimsson et al. 2002). Changes in morphology are influenced by interactions among different parts of the organism (i.e., modularity; Wagner and Altenberg 1996; Hallgrimsson et al. 2002) and are shaped by the confluence of development and function, some as adaptive responses to the environment (e.g., Olsen and Miller 1951; Marroig and Cheverud 2001; Piras et al. 2010; Goswami et al. 2014). The evolution of monodactyly in horses (Perissodactyla, Equidae, *Equus* Linnaeus, 1758) is perhaps one of the best-known examples of morphological change as an adaptation to the environment (Hildebrand 1987; Alexander 1998; Currey 2002; McHorse et al. 2017). The ancestral condition for equids is four toes on the forelimbs and three toes on the hindlimbs, as exhibited by *Hyracotherium* Owen, 1841 (MacFadden 1994). The evolution of monodactyly in equids began with a shift towards unguligrade (more upright) foot posture, involving the elongation of metapodial III, lengthening of tendons, and a proximal concentration of force-generating musculature (Clayton 2016; Janis and Bernor 2019). This is thought to have been a response to the shift from a forested habitat to grasslands; hard substrates may select for long, slim legs to increase speed for predator escape (Simpson 1951), decrease the energetic costs of movements by reducing distal limb mass (Janis and Wilhelm 1993), increase efficiency for long-distance travel to find food (Janis and Bernor 2019), and enhance stability for rapid, unidirectional movements (Shotwell 1961). The evolution of large body mass among horses may also have increased the bending forces on limbs, selecting for a single toe because one digit resists bending forces better than multiple smaller digits of the same overall size (Thomason 1986; Biewener 1998; McHorse et al. 2017).

The post-cranial skeletal anatomy of *Equus* may represent a strongly integrated system due to phylogenetic constraints, resulting in relative morphological homogeneity among species (Biewener and Patek 2018; Hanot et al. 2018). As such, there may be little evolvability within the post-cranial skeleton, and shape changes may be highly restricted. Metapodials have thus been considered one of the most useful skeletal elements for identifying ancient species of *Equus* (Winans 1989). However, modern domestic horses (*Equus caballus* Linnaeus, 1758) show variability in limb bone morphology and structural properties as a result of artificial selection. For example, selective breeding of Thoroughbred horses for racing has resulted in longer, more slender limb bones compared to other breeds and in limbs that operate on anatomical and biomechanical extremes (Alexander 1998; Currey 2002; Goldstein et al. 2021). Further, domestic horses vary greatly in size (Brooks et al. 2010), which impacts the shape of the limb bones; smaller horses, such as Icelandic horses and Shetland ponies have smaller, more slender limb bones, while larger horses, such Clydesdales and other draft breeds, possess larger, very robust limb bones (Hanot et al. 2018). It is therefore possible that, over evolutionary time, natural selection also produced variability in distal limb bone morphology of non-domesticated horses.

Many extinct species of *Equus* have been named based on morphological characteristics such as metapodial morphology, some having been named based solely on fragmentary fossil remains (e.g., *Equus occidentalis* in Leidy 1865; *Equus semplicatus* in Cope 1893; *Equus giganteus* in Gidley 1901; *‘Equus’ francisci* in Hay 1915). Several horse species (*n* = 6) have been named from Beringia (present-day Siberia, Alaska, and Yukon), but the validity of these species has recently been questioned (e.g., Weinstock et al. 2005; Barrón-Ortiz et al. 2017; Heintzman et al. 2017; Vershinina et al. 2021). Weinstock et al. (2005) suggested that there existed only two lineages of horses in North America during the late Pleistocene, the caballine (or stout-legged horses) belonging to the genus *Equus* and the stilt-legged horses belonging to the genus *Haringtonhippus* Heintzman et al., 2017 (although this genus is debated), and that each lineage may have been comprised of a single, wide-ranging species. Vershinina et al. (2021) stated that, based on recent genomic evidence, there is insufficient support for distinct taxonomic groups and that some of the named Beringian horse species are likely *nomina nuda* (empty names) despite their correctly formed nomenclature. Further, Guthrie (2003) suggested that horses underwent a reduction in body mass to cope with the warming climate of the Late Pleistocene (~ 37–12.5 ka), thus it is not out of the question that changes in body mass created a scenario where multiple invalid species were named.

Here, we analyze the third metapodials (metatarsals) from four Pleistocene horse species that were proposed to have coexisted in Beringia to test whether there were significant differences in metapodial morphology that would support the existence of multiple species. We hypothesize that, based on the findings of Heintzman et al. (2017) there will be two distinct groups, each most likely comprised of a single species: the caballine (stout-legged) *Equus* group, and the stilt-legged *Haringtonhippus* group. If we can quantitatively distinguish between metapodials pertaining to *Equus* and *Haringtonhippus* but not those assigned to species within *Equus*, then it is most likely that there is indeed only one species belonging to each genus. Alternatively, if we find that metapodials can be separated into distinct groups pertaining to the assigned species, then there exists the possibility for the co-existence of multiple horse species.

## Materials and methods

In total, 203 hind-leg metapodials (metatarsals) were obtained from the Quaternary Zoology collections at the Canadian Museum of Nature. The selected metapodials are not associated with any other skeletal material and are presumed to represent individual animals. We relied on the previous species identifications for this study and did not perform any identifications ourselves. The selected metapodials represent four identified species: *Equus lambei* Hay, 1917 (*n* = 103), *Equus scotti* Gidley, 1900 (*n* = 8), *Equus verae* Sher, 1971 (*n* = 18), and *Haringtonhippus francisci* (catalogued as *Equus* (*Asinus*) cf. *kiang* Moorcroft, 1841; *n* = 7), as well as metapodials that were not identified to the species level, referred to as *Equus* sp. (*n* = 67). All metapodials were collected from the Yukon Territory (largely from the Dawson and Old Crow regions), which was part of eastern Beringia during the Late Pleistocene (> 54 –11.7 ka). Specimens were selected based on the completeness of the metapodial; small breaks were acceptable, but metapodials that were severely broken or fragmented were excluded.

**Fig. 1:**
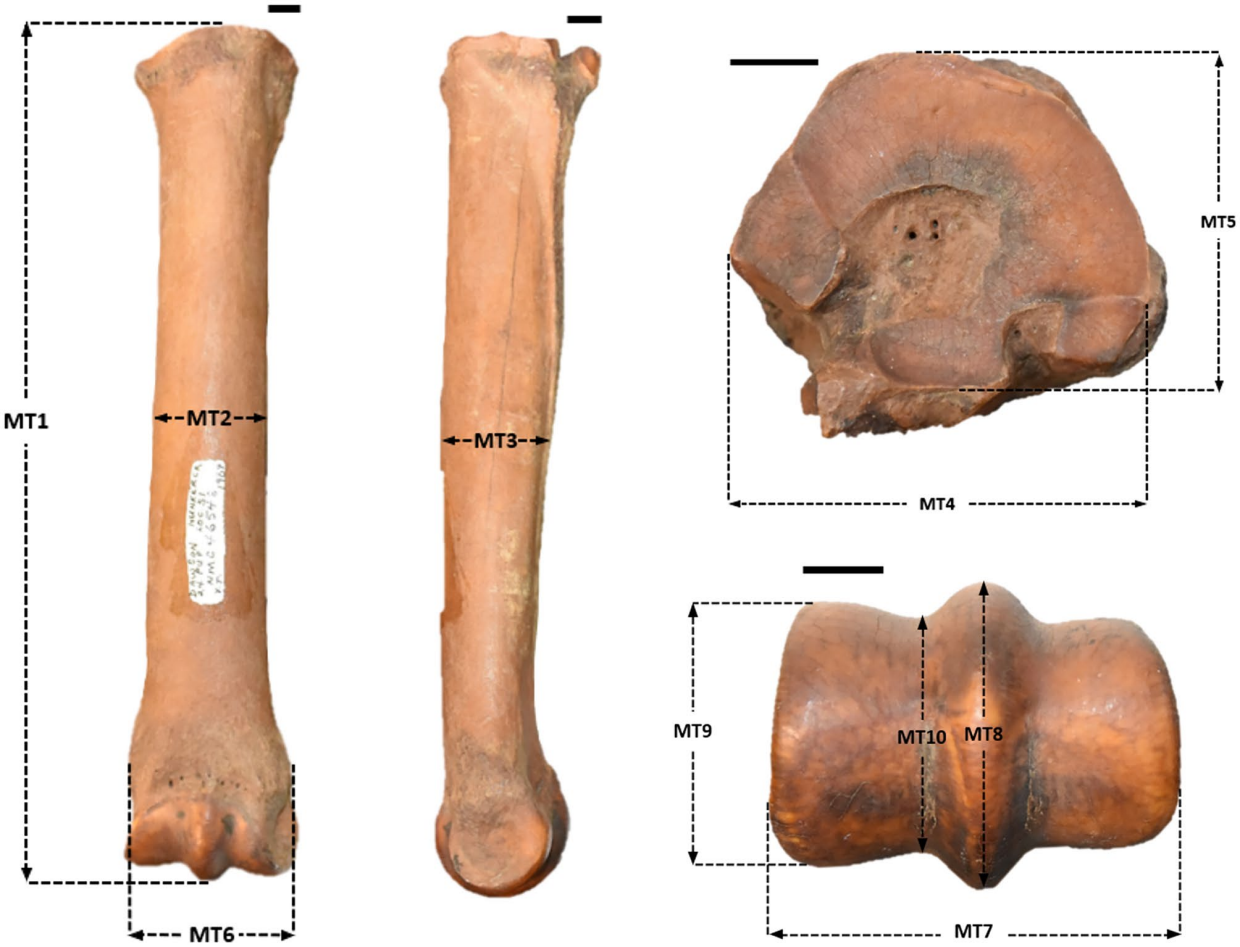
Metapodial measurements illustrated on the left metatarsal of CMNFV 46548 (*Equus* sp.). Measurements are: MT1, greatest length; MT2, smallest mid-shaft width; MT3, depth of diaphysis; MT4, proximal articular breadth; MT5, proximal articular depth; MT6, distal maximal supra-articular breadth; MT7, distal maximal articular breadth; MT8, distal maximal depth of keel; MT9, distal maximal depth of medial condyle; MT10, distal minimal depth of medial condyle. Scale bars each represent 1 cm

Ten measurements, all of which are as described by Eisenmann (1986) and seven of which are described by Winans (1989), were taken for each metapodial using digital calipers (accuracy 0.01 mm). All measurements reported are in millimeters (Online Resource 1). We took the following measurements on each specimen, unless the specimen was broken at a given location (numbers in brackets refer to the corresponding measurements used by Eisenmann (1986) and Winans (1989), respectively): MT1, greatest length (1,1); MT2, smallest/mid-shaft width (3, 6); MT3, depth of diaphysis (level of three; 4, -); MT4, proximal articular breadth (5, 2); MT5, proximal articular depth (6, 3?); MT6, distal maximal supra-articular breadth (10, 8); MT7, distal maximal articular breadth (11, 9), MT8, distal maximal depth of keel (12, -); MT9, distal maximal depth of medial condyle (14, 10); and MT10, distal minimal depth of medial condyle (13, -) (Fig. 1). Orientation (left or right) for each metapodial was also noted.

Specimens that were missing one or more measurement value (*n* = 21) were excluded from statistical analyses. All R code used in this study is available in Online Resource 2. We log transformed the metapodial measurements and applied a principal component analysis (PCA) in the R software environment using the factoextra package (Kassambara and Mundt 2020; R Core Team 2021) to reduce the dimensionality of the dataset and to visualize how individuals are distributed in the morphometric space (i.e., to determine whether there are any trends, clusters, or outliers in the data). We also performed a linear discriminate analysis (LDA) in R using the MASS, calibrate, ggforce, and concaveman packages (Gombin et al. 2020; Graffelman 2020; Pedersen 2021; R Core Team 2021; Ripley et al. 2022) on the metapodial measurements from specimens that were previously assigned to a species to test the given species classifications. We then used the LDA results and Bayesian inference to assign the unknown (*E*. sp.) specimens to one of the four identified species groups using equally weighted priors (Ripley et al. 2022).

Finally, we reconstructed the body mass of every specimen using linear regressions from Scott (1990) and Alberdi et al. (1995) that correspond to our metapodial measurements. We used six of the seven regressions from Scott (1990) and nine of the fourteen regressions from Alberdi et al. (1995). The body masses that were reconstructed using the regression that was the best predictor of body mass (i.e., the regression with the highest *R*^2^ value) from each paper were used to conduct one-way ANOVAs in R using the dplyr and ggpubr packages (Kassambara 2020; R Core Team 2021; Wickham et al. 2022) to determine whether mean body mass differed among the four identified species groups and the specimens without a confirmed species identification. Measurement MT7 (distal maximal supra-articular breadth; *R*^2^ = 0.8526) was used from Scott (1990; Eq. 1; referred to as MT4 in Scott 1990), and MT8 (distal maximal depth of keel; *R*^2^ = 0.9467) was used from Alberdi et al. (1995; Eq. 2; referred to as MT12 in Alberdi et al. 1995) (Fig. 1).

1 $${\mathrm{Log}}_{10}\left(\mathrm{Body\ Mass}\right)=\left[2.4247\times {\mathrm{Log}}_{10}\left(\mathrm{MT}7\right)\right]+0.8911$$

2 $$\mathrm{LN}\left(\mathrm{Body\ Mass}\right)=\left[2.768\times \mathrm{LN}\left(\mathrm{MT}8\right)\right]+(-4.061)$$

Following the ANOVAs, Tukey’s HSD tests were performed to determine (if there were significant differences among groups) which groups differed significantly from one another.

## Results

**Fig. 2:**
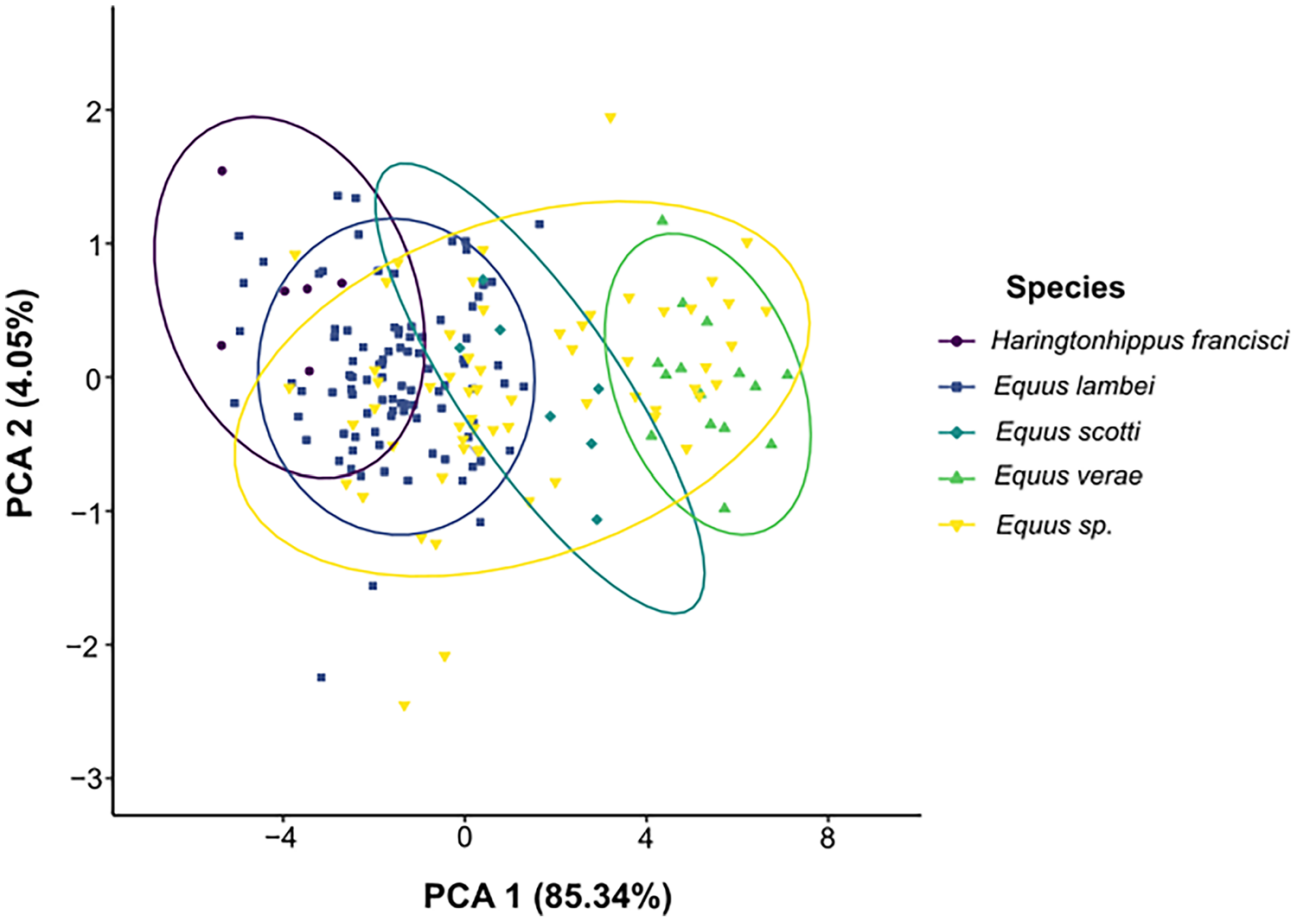
Morphospace of Beringian horse metapodials based on principal component analysis and grouped by species, with 95% confidence ellipses

Much of the proportion of variance in the metapodial dataset was explained by PC1 (85.34% ± 2.92) (Fig. 2; Online Resource 3). In contrast, PC2 explained a small proportion of the variance (4.05% ± 0.64), and PC3 explained even less of the variance (2.84% ± 0.53) (Online Resource 3). Due to the very small contribution of PC3 to the cumulative variance, we do not consider it to be of notable importance and do not discuss it further. PC1 loads most heavily on the distal maximal articular breadth; however, all the variables are strongly positively correlated with one another (Online Resource 4). Thus, we consider PC1 to represent variation associated with overall metapodial size. We observe discernible grouping between the four identified species groups, although there is gradation in morphospace along PC1 (Fig. 2). Of the four defined species groups, the specimens attributed to *E. lambei* exhibited the greatest range in metapodial measurements and shared the morphospace with all but one species group (*E. verae*), with the majority of specimens falling slightly to the left of the midpoint of PC1, indicating that they overall tended to have smaller metapodials (Fig. 2). The *E. scotti* group was fairly centralized on PC1 but exhibited notable overlap with the *E. lambei* group, indicating that specimens from the *E. scotti* group have comparable but typically somewhat larger metapodials than those assigned to *E. lambei* (Fig. 2). The *E. verae* group overlaps the least with the other defined species groups, only slightly sharing the morphospace of PC1 with the larger end of the *E. scotti* group (Fig. 2). The stilt-legged horse group, *H. francisci*, exhibited some of the smallest metapodials but also shared the morphospace with *E. lambei* and, to a lesser extent, *E. scotti*. When considering the indeterminate species group (*E*. sp.), we observe overlap in morphospace with all other species groups, representing some of the smallest and largest individuals within the entire dataset (Fig. 2).

Although the variation explained by PC2 is small, it is still notable because it loads strongly on the depth of diaphysis (i.e., anterior–posterior width) and produces two somewhat discernible groupings: the stilt-legged horse (*H. francisci*) and the stout-legged horses (all *Equus* groups) (Fig. 2). However, much like PC1, there exists considerable overlap between species groups, most notably between the *H. francisci* and *E. lambei* groups. Although most *E. lambei* specimens plot close to 0 and within close proximity to the other stout-legged horse species groups (Fig. 2), several *E. lambei* specimens plot more closely to stilt-legged group along PC2, indicating that these *E. lambei* specimens exhibit more slender metapodials similar to those of *H. francisci*; interestingly, there are also specimens at the opposite end of the axis, suggesting that these individuals had more robust metapodials. Intriguingly, although most of the *E. scotti* specimens plot around 0 on PC2, the overall *E. scotti* morphospace is considerably larger along PC2 than either of the other two discrete *Equus* groups, with individuals tending towards the more stout-legged morphology (Fig. 2). This could simply be the result of a smaller sample size, but we do not discount the potential for this to be the result of plasticity of metapodial morphology in the genus *Equus* as a whole. Most of the *E. verae* specimens also plotted close to 0, similar to the other stout-legged horses, although there were a few individuals that deviated from this trend (Fig. 2). Much like PC1, the *E.* sp. specimens plot throughout the entire morphospace of PC2 (Fig. 2), alongside stilt-legged and stout-legged horse groups alike.

The LDA correctly classified species based on the metapodial measurements 95.09% of the time (misidentification rate was 4.91%; full loadings available in Online Resource 5). Both *H. francisci* and *E. verae* specimens were correctly identified 100% of the time, *E. lambei* specimens were correctly identified 95.74% of the time, while *E. scotti* specimens were correctly identified 71.43% of the time (Table 1). The specimens that were designated *E.* sp. were all re-classified into one of the four identified species groups; 27 specimens were assigned to *E. lambei* (44.26%), 20 specimens were assigned to *E. verae* (32.79%), 12 specimens were assigned to *E. scotti* (19.67%), and two specimens were assigned to *H. francisci* (3.28%; Table 1). Most of the *E*. sp. specimens being separated into either the *E. lambei* or *E. verae* group was unsurprising, considering that they not only appear to be more common in the fossil record but also are clearly distinct from one another within the PCA morphospace. We consider it interesting that the LDA assigned comparatively few *E*. sp. specimens to *E. scotti* (Table 1). This could be an artifact of the small sample size of *E. scotti* in our dataset, but we argue that this is more likely due to the intermediate metapodial size exhibited by *E. scotti,* which falls between two well-represented and distinct species groups (*E. lambei* and *E. verae*). It is difficult to differentiate *E. scotti* from either of these species groups, particularly *E. lambei*, due to the high variation exhibited in the metapodial size of *E. lambei* group. The low assignment of *E.* sp. specimens to the *H. francisci* group was also unexpected, considering that *H. francisci* is the only stilt-legged horse represented in our dataset and present in eastern Beringia. This finding could simply be due to the relative abundance of *H. francisci* specimens compared to other species, as it is possible that stilt-legged horses were less common than their stout-legged counterparts and left behind fewer fossil remains, thus limiting our sample size. It is also possible that, due to the suggested plasticity of stilt-legged horse metapodials (Barrón-Ortiz et al. 2017) and the extensive overlap in metapodial size with the well-represented *E. lambei* that we are unable to properly distinguish between stilt-legged horses and smaller, more gracile morphs of stout-legged horses.

**Table 1:** Classification table of Beringian horse species from the LDA

	*Haringtonhippus francisci* (*n* = 6)	*Equus lambei* (*n* = 94)	*Equus scotti* (*n* = 7)	*Equus verae* (*n* = 15)	*Equus *sp. (*n* = 61)
*Haringtonhippus francisci*	6	3	0	0	2
*Equus lambei*	0	90	2	0	27
*Equus scotti*	0	1	5	0	12
*Equus verae*	0	0	0	15	20

**Fig. 3:**
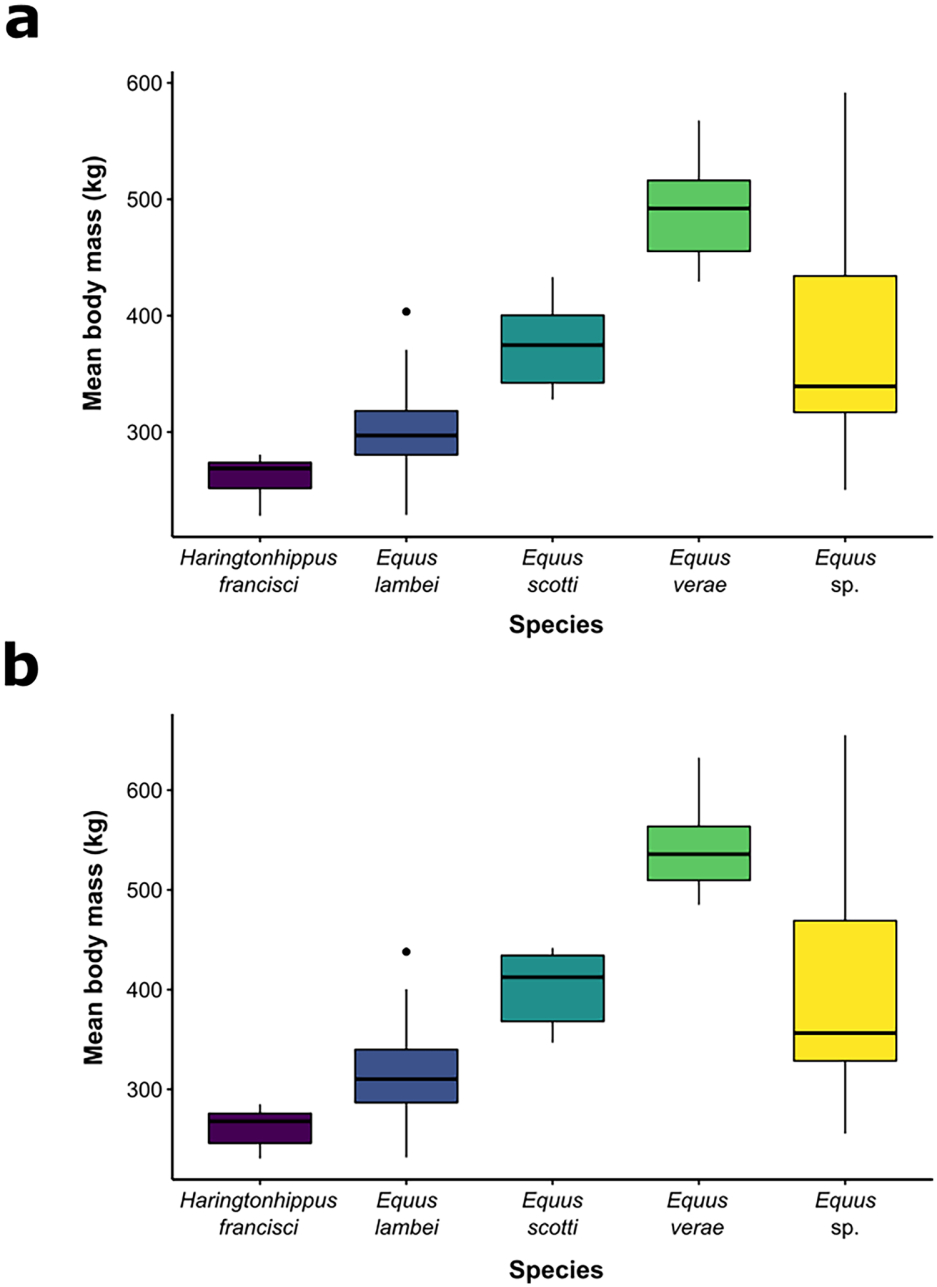
Estimated body masses of Beringian horses. **a**. Calculated from MT4 using the equation from Scott et al. (1990); **b**. Calculated from MT8 using the equation from Alberdi et al. (1995)

The regression from Alberdi et al. (1995) (Fig. 3b) produced slightly larger body mass estimates than the estimates obtained using the equation from Scott (1990) (Fig. 3a), and full body mass estimates are available in the Supplementary Information (Online Resource 6, 7). Based on our body mass reconstructions, *H. francisci* is the smallest, *E. lambei* and *E. scotti* are mid-sized, and *E. verae* is the largest by a considerable margin (> 100 kg) (Fig. 3; Table 2). The specimens catalogued as *E*. sp. had the overall widest range of body mass estimates, which is unsurprising considering that they likely belong to any one of the four identified species groups (Fig. 3; Table 2). There was a significant difference in body mass among the groups for both the masses reconstructed from Scott (1990) (ANOVA, *F* = 61.49, *df* = 4, *SS* = 703,248, *p* < 0.001) and Alberdi et al. (1995) (ANOVA, *F* = 60.97, *df* = 4, *SS* = 993,767, *p* < 0.001). The only groups that are not significantly different from one another are the *E. scotti* and *E.* sp. groups, and the *E. lambei* and *H. francisci* groups, respectively (Table 3).

**Table 2:** Estimated body masses (in kg) of each species reconstructed from MT6 (Scott 1990) and MT8 (Alberdi et al. 1995)

	(Scott 1990)		(Alberdi et al. 1995)	
*Taxa*	Range	Mean	Range	Mean
*Haringtonhippus francisci*	175.53 – 229.27	210.44 ± 16.94	201.93 – 278.40	227.72 ± 25.63
*Equus lambei*	202.30 – 395.93	295.01 ± 39.72	181.95 – 483.04	326.01 ± 53.44
*Equus scotti*	294.34 – 436.19	374.16 ± 47.86	342.07 – 466.04	422.57 ± 40.96
*Equus verae*	407.23 – 573.32	492.59 ± 45.15	495.04 – 694.15	573.49 ± 56.34
*Equus *sp.	197.10 – 565.33	365.31 ± 89.13	239.37 – 631.06	416.83 ± 101.02

**Table 3:** Results of the Tukey’s HSD post-hoc tests conducted on the estimated body masses

	(Scott 1990)		(Alberdi et al. 1995)	
	Mean difference	p-value	Mean difference	p-value
*lambei – francisci*	37.543	0.378	51.768	0.234
*scotti – francisci*	112.390	< 0.001*	141.105	< 0.001*
sp.* – francisci*	110.063	< 0.001*	134.980	< 0.001*
*verae – francisci*	229.619	< 0.001*	280.717	< 0.001*
*scotti – lambei*	74.847	< 0.005*	89.337	< 0.005*
sp.* – lambei*	72.521	< 0.001*	83.212	< 0.001*
*verae – lambei*	192.077	< 0.001*	228.949	< 0.001*
sp.* – scotti*	–2.326	1.000	–6.125	0.999
*verae – scotti*	117.229	< 0.001*	139.612	< 0.001*
*verae – *sp.	119.556	< 0.001*	145.737	< 0.001*

## Discussion

The size and morphology of equid metapodials have been used in multiple studies to infer taxonomic identity (e.g., Winans 1989; Baskin and Mosqueda 2002; Bernor et al. 2003; Weinstock et al. 2005; Alberdi et al. 2014; Barrón-Ortiz et al. 2017; Heintzman et al. 2017; Marín-Leyva et al. 2019; Sun et al. 2022). Here we show that, based on the results of our PCA, there is extensive overlap among the species groups along both axes, with individuals from the indeterminate species group plotting with all four named species groups. We also provide novel body mass estimates for Beringian equids that demonstrate that there is a continuous gradation in body mass among the named horse species, both including and excluding the *E*. sp. specimens. Although the LDA appears to distinguish among identified species groups, due to continuous variation in body size and, therefore, metapodial size among the groups (including *E.* sp.), we do not consider metapodial measurements to be ideal taxonomic indicators for Beringian equids and suggest that the results of the LDA are based entirely on metapodial size. Due to the possibility for plasticity in the morphology of equid metapodials, the defined species groups could simply reflect different size morphs of Beringian horses that may only encompass one or two species, as previous work has suggested (Weinstock et al. 2005; Heintzman et al. 2017; Vershinina et al. 2021).

Effectively, much of the variation that is present within our dataset is likely explained by the continuous variation in metapodial size, and therefore the continuous variation in body mass. Even when the *E*. sp. specimens are not considered, there is a clear overlap among named species groups in the PCA morphospace (especially along PC1) that appear to reflect a gradation of metapodial size among Beringian horse species (Fig. 2). For example, we observe that *H. francisci* have the smallest and most slender metapodials, whereas *E. verae* have larger, generally robust metapodials; *E. lambei* and *E. scotti* are more intermediate in metapodial size, although *E. lambei* tends towards the smaller size and *E. scotti* towards the larger (Online Resource 1). When we consider the *E.* sp. specimens, we see this same trend of continuous variation in metapodial size is also represented, with *E.* sp. specimens falling within the morphospaces of all species groups (Fig. 2). Since metapodials are reliable predictors of body mass (Scott 1990; Alberdi 1995; Mendoza et al. 2005) and overall metapodial size loads strongly on PC1 (Fig. 2), the gradation in metapodial size appears to be strongly related to body mass.

Body mass alone exerts one of the strongest, if not the strongest, control on limb bone morphology (Hildebrand 1982; Biewener 1989; Polly 2008). Bending and compression forces increase in proportion to an animal’s mass (Etienne et al. 2020) and the limb bones must withstand these stresses. The ability of bones to resist such forces depends on their cross-sectional area (Biewener 1989). The evolution of unguligrade posture and monodactyly in horses were key adaptations that allowed for increased in body mass throughout their evolutionary history (McHorse et al. 2017). However, above a certain mass (~ 300 kg; Biewener 1989, 2005) there exists a threshold where it becomes challenging for the limb bones to become any more upright in their position. To compensate for the increase in forces, the shape of the limb bones will often undergo more extreme changes in morphology (Biewener 1989; Bertram and Biewener 1990; Christiansen 1999). Typically, the most obvious change in limb bone morphology as body mass increases is an increase in overall robustness (i.e., an increase in diameter relative to the length; Schmidt-Nielsen 1984).

Modern domestic horses, which vary more than twofold in body size (Brooks et al. 2010), exhibit considerable phenotypic variability. For example, draft horses such as Shires and Clydesdales have very large, robust limb bones to support their heavy body mass, whereas smaller horses such as Icelandic horses and Shetland ponies possess smaller, more gracile limb bones (Hanot et al. 2018). Even horse breeds that are used to perform similar tasks can differ greatly from one another in their limb morphology; Thoroughbreds have much longer and more slender metapodials than Quarter Horses, though both breeds are frequently used in racing (Goldstein et al. 2021). Guthrie (2003) and Barrón-Ortiz et al (2017) proposed that the metapodial morphology of extinct horses were also plastic, highlighting variation in metapodial shape for *E. lambei* and *H. francisci*, respectively. Our findings of continuous variation in both metapodial size and body mass support the hypothesis that there was notable metapodial plasticity exhibited by Beringian equids. The possibility for plasticity in metapodial and body size, coupled with the influence of body mass on metapodial morphology, thus limits the utility of metapodials as taxonomic indicators for Beringian horses. Taxonomic quality varies with body mass, with large-bodied mammals tending to be severely overspilt relative to smaller mammals, a problem that is particularly evident in perissodactyls (Alroy 2003). While we do not make any claims regarding the validity of specific taxa here, we do encourage the use of other techniques to identify *Equus* species (e.g., palaeogenomics), and to determine features that are more clearly distinguishable between stilt- and stout-legged horses, as the phylogenetic relationship between these groups is among the most contentious and poorly resolved (Weinstock et al. 2005; Eisenmann et al. 2008; Barrón-Ortiz et al. 2017, 2019; Heintzman et al. 2017; Priego-Vargas et al. 2017; Jiménez-Hidalgo and Díaz-Sibaja 2020).

Although identifying drivers of variation in metapodial morphology among Beringian horses is outside the scope of this study, we provide some suggestions regarding possible drivers and encourage further investigation. There is increasing evidence that global climate changes affect animal body mass (Gardner et al. 2011; Sheridan and Bickford 2011; Secord et al. 2012; Martin et al. 2018), although species can differ in the direction, rate, and extent of body mass change (Lovegrove and Mowoe 2013). Large mammals, in particular, show rapid declines in body mass as temperatures rise (Evans et al. 2012), in line with Bergmann’s rule (Bergmann 1847; Ashton et al. 2000; Freckleton et al. 2003; Rodríguez et al. 2008). A number of large mammals similarly underwent changes in body size in response to climatic shifts, specifically glacial to interglacial cycles, during the Pleistocene (Guthrie 1982; Noguéz-Bravo et al. 2008; Van Asperen 2010; Zimov et al. 2012; Raghavan et al. 2014; Rasmussen et al. 2014; Martin et al. 2018; Pineda-Munoz et al. 2021). For example, the size of *E. lambei* appears to vary in response to climatic changes. Guthrie (2003) demonstrated that, in Alaska, *E. lambei* underwent a drastic reduction in body size (~ 15%) as a response to the warming climate following the Last Glacial Maximum and into the Holocene, before ultimately going extinct in North America ~ 12.5 ka. Given that the *Equus* taxa here likely lived during several climate changes, it is possible that metapodial morphology reflects body mass changes through time rather than interspecific morphological differences. Furthermore, our present analysis lacks temporal context. We are thus unable to investigate the potential relationship between climate change and metapodial size, and subsequently body mass, but we encourage future studies to pursue this relationship with the inclusion of novel radiocarbon dates.

In addition to climate, metapodial morphology is influenced by species ecology. Metapodial morphology has been linked with habitat preference in bovids (Köhler 1993; Plummer and Bishop 1994; Mendoza and Palmqvist 2006) and equids (Schellhorn and Pfretzschner 2015; Li et al. 2021). The presence of a single metapodial is generally thought to be an adaptation to more open habitats, such as grasslands or tundra (Köhler 1993; Scott 1985; McHorse et al. 2017). Beringian horses inhabited the mammoth steppe, a megacontinental ecosystem characterized by the presence of megafauna that persisted from ~ 115–11.7 ka (Guthrie 1982; Drucker 2022). The northern Yukon Territory was part of the mammoth steppe and acted as a glacial refugium for a multitude of northern North American species because the climate was too dry to permit extensive glaciation (Zazula et al. 2006; Froese et al. 2009; Zimov et al. 2012). The mammoth steppe was a predominantly treeless, steppe-tundra landscape dominated by grasslands composed of cold- and dry-adapted vegetation (Hibbert 1982; Schweger 1982; Zimov et al. 1995; Zazula et al. 2003). Eventually, the arid mammoth steppe was progressively replaced by boreal forest during the Late Pleistocene–Holocene transition (~ 11.7 ka), which could potentially explain the gradation observed in metapodial size of Beringian horses; more closed habitats tend to select for smaller body mass and, by association, smaller metapodials (Köhler 1993; Schellhorn and Pfretzschner 2015). Unfortunately, due the lack of radiocarbon dates, we cannot determine whether the continuous variation in our dataset represents a change in size within a species over time.

We cannot directly assess the validity of the various named Beringian horse species here but have uncovered evidence that there is separation by body mass that could be unrelated to phylogenetic position, though we cannot yet reject the coexistence of multiple species. In fact, the mammoth steppe ecosystem is considered to have been a highly productive environment capable of supporting many species of large herbivores (Guthrie 1982, 2001; Zimov et al. 2012; Willerslev et al. 2014; Zhu et al. 2018), including species with overlapping niches (Bocherens 2003; Pires et al. 2015; Davis 2017), not unlike the African savannah. There are several species of modern equids that co-exist in Africa with many other species of large herbivores (i.e., elephants, wildebeasts, antelope) by niche partitioning (e.g., Voeten and Prins 1999; Cromsigt and Olff 2006; Schulz and Kaiser 2013; Kartzinel et al. 2015; Mandlate et al. 2019) that is driven in part by differences in body mass among species (Kleynhans et al. 2011). Beringian equids would have similarly co-existed with other megaherbivores such as mammoths, bison, and muskox (Harington and Clulow 1973; Harington 1980, 1990, 2011; Hughes et al. 1981; Weber et al. 1981; Porter 1986), likely by some degree of niche partitioning behaviours (e.g., Guthrie 2001; Fox-Dobbs et al. 2008; Schwartz-Narbonne et al. 2019; Drucker 2022). Although palaeoecological research on Beringian equids is limited, they are thought to have been primarily grazers that occasionally consumed some browse vegetation (Guthrie 2001; Fox-Dobbs et al. 2008; Semprebon et al. 2016; Kelly et al. 2021). In other regions, ancient equids are believed to have partitioned resources based on their sizes; larger horses consumed tall, coarse grasses and more browse vegetation, while the smaller horses were predominantly grazing on shorter, softer grasses (Van Asperen 2010; Wolf et al. 2010; Saarinen et al. 2021). We therefore cannot reject the possibility that multiple species of Beringian horse did co-exist, doing so via partitioning dietary resources. Based on the findings of previous research on size-based niche partitioning in horses (Van Asperen 2010; Wolf et al. 2010; Saarinen et al. 2021), we suggest that the smaller morphs (i.e., *H. francisci* and *E. lambei*) would likely have been primarily grazers, while the larger morphs (i.e., *E. scotti* and *E. verae*) were probably mixed feeders and incorporated both grass and browse in their diets. However, this is purely speculative as we do not incorporate any dietary indicators (i.e., stable isotope analysis, dental microwear/mesowear) or ecological niche modelling in the present study, but we encourage future studies to investigate the dietary ecology of Beringian horses to elucidate whether niche partitioning may have been a mechanism by which multiple species could have co-existed.

## Conclusion

The metapodials of extinct horses have long been used as sources of taxonomic data. Here, we analyzed the metapodials of 203 fossil horse specimens from Beringia to determine the reliability of these skeletal elements as taxonomic indicators. We find that the four identified horse species are distinguished from one another based almost entirely on overall metapodial size, which is an unreliable indicator of taxonomy because there exists variability in size both within and among species that can be influenced by environmental changes. Mean body mass for several of the included species differ significantly, but there remains considerable overlap in body mass estimates among several species (*E. lambei* and *H. francisci*, *E. scotti* and *E*. sp.). The continuous variation in metapodial size and robusticity exhibited by the *E*. sp. specimens further highlights that metapodial morphology was likely plastic in ancient horses, and that metapodials are not reliable indicators of taxonomy taken on their own. Metapodial morphology in Beringian horses may also have changed over time as a result of climate change, but we do not possess the requisite radiocarbon dates to test this hypothesis. We therefore suggest that metapodial morphology cannot differentiate between a single species with considerable body size variation, several species that differ along a body mass spectrum but did not coexist temporally, and several differently-sized species that did coexist via dietary niche partitioning. Regardless, the unresolved identity and true number of Beringian horse species inhibits our understanding of ecosystem dynamics of the mammoth steppe, and reconciling the taxonomy is key to furthering our understanding of the extinction cause for the different Beringian horse species at the end of the Pleistocene. We encourage future studies aimed at resolving the taxonomy of these horses to avoid the use of metapodials and instead to use more reliable indicators of taxonomy, such as cheek tooth morphology or palaeogenomics, alongside accurate radiocarbon dates to uncover the phylogenetic relationships among and the true number of Beringian horse species.

## Supplementary Information

Below is the link to the electronic supplementary material.

Supplementary file1 (XLSX 29 KB)

Supplementary file2 (PDF 209 KB)

Supplementary file3 (XLSX 10 KB)

Supplementary file4 (XLSX 11 KB)

Supplementary file5 (XLSX 18 KB)

Supplementary file6 (XLSX 23 KB)

Supplementary file7 (XLSX 27 KB)

### Supplementary Information

Below is the link to the electronic supplementary material.

## Data Availability

All data generated or analyzed during this study are included in this published article and its supplementary information files and are available from the corresponding author upon request.
