# Supplementary material for: Investigating the reliability of metapodials as taxonomic Indicators for Beringian horses
Source: J Mamm Evol. 2022 Sep 21;29(4):863–75. doi: 10.1007/s10914-022-09626-4 (PMC9684255; doi:10.1007/s10914-022-09626-4)
Supplement: Supplementary file 2 — Supplementary file2 (PDF 209 KB) [file 10914_2022_9626_MOESM2_ESM.pdf]

Online Resource 2: R Code

INVESTIGATING THE RELIABILITY OF METAPODIALS AS TAXONOMIC INDICATORS FOR BERINGIAN HORSES

Zoe Landry[1,2], Mathew J. Roloson[2,3], Danielle Fraser[2,3,4,5]

[1]Department of Earth Sciences, University of Ottawa, 25 Templeton Street, Ottawa, Ontario K1N 6N5, Canada

[2]Beaty Centre for Species Discovery, Canadian Museum of Nature, PO Box 3443 Stn "D", Ottawa, ON K1P 6P4, Canada

[3]Department of Earth Sciences, Carleton University, 1125 Colonel By Drive, Ottawa, Ontario K1S 5B6, Canada

[4]Department of Biology, Carleton University, 1125 Colonel By Drive, Ottawa, Ontario K1S 5B6, Canada

[5]Paleobiology, Smithsonian National Museum of Natural History, Washington, DC 20560, United States of America

Corresponding author: Zoe Landry zland032@uottawa.ca

ORCID: Zoe Landry, 0000-0002-5527-9454; Danielle Fraser, 0000-0002-0228-2617

Principal component analysis

```
library(factoextra)
library(ggplot2)
library(viridis)

setwd("~/Metapodials project")

data<-read.csv("metapodials_pca_log.csv", header = TRUE)
data<-na.omit(data)
data

PCA<-
prcomp(~Log10_Greatest_Length+Log10_Smallest_Width+Log10_Depth_of_diaphysis+Log10_Prox_articular_breadth+Log10_Prox_articular_depth+Log10_Dist_max_supra_articular_breadth+Log10_Dist_max_articular_breadth+Log10_Dist_max_depth_of_keel+Log10_Dist_max_depth_medial_condyle+Log10_Dist_min_depth_medial_condyle, data = data, scale=T)
biplot(PCA, cex=0.5, pch=2)

forPlot<-data.frame(PCA$x, data$Species)
loadings<-as.data.frame(PCA$rotation)
loadings$Names<-rownames(loadings)
summary(PCA)

res.var <- get_pca_var(PCA)
res.var$contrib

ggplot(forPlot, mapping=aes(x=PC1,y=PC2)) +
  geom_segment(data=loadings, aes(x=0, y=0, xend=PC1, yend=PC2),
arrow=arrow(length=unit(0.2,"cm")), alpha=0.25)+
  geom_text(data=loadings, aes(x=PC1, y=PC2, label=Names), alpha=0.5, size=3)+
  geom_point(mapping=aes(shape=data$Species,colour=data$Species, fill=data$Species),size=1) +
  scale_shape_manual(values = c(21,22,23,24,25)) +
  scale_color_viridis(discrete = TRUE, option = "D") + scale_fill_viridis(discrete = TRUE) +
  theme_classic() + stat_ellipse(aes(colour=data$Species)) + xlim(-7,8) + ylim(-3,2.5)

#clustering

PCA1<-prcomp(PCA$x, center=FALSE, scale=FALSE, rank = 4) # stats::
results <- PCA1
hopkins(df, n=nrow(results)-1)
```

Linear discriminant analysis

```
library("MASS")
library("calibrate")
library("ggforce")
library("concaveman")
library(“Rtools”)

dat<-read.csv("metapodials_lda_part_log.csv", header = TRUE, row.names = 1)

#prep data for prediction, remove unknowns (sp.)

dat.known <-dat[-c(108:168),]
dat.unknown <- dat[-c(1:107,169:183),]

dat.known$Species

#run LDA

LDA <- lda(Species~., data=dat.known)
ldpred <- predict(LDA)
ld1 <- ldpred$x[,1]
ld2 <- ldpred$x[,2]
ld3 <- ldpred$x[,3]
#write.csv(ldpred$x, "LDAscores2.csv")

ld1

dat.known.drop

confusion <- table(predict(LDA)$class, dat.known$Species)
confusion

predict <- data.frame(ldpred["class"]) # Pull functional predictions
predict

misid <- 1-(sum(diag(confusion))/sum(confusion)) # Find the misidentification rate
misid

scaling <- (LDA["scaling"])
scaling <- data.frame(matrix(unlist("scaling"), nrow = #?, byrow=T))
scaling <- t("scaling")

dat.unknown2 <- dat.unknown[-c(1)]
dat.unknown2

dat.unknown
```

```
#predict unknown taxa
priors <- c(0.25,0.25,0.25,0.25)
assigns <- predict(LDA, dat.unknown2, prior=priors)
assigns
assigns$class

#plotting the LDA

plot(ldpred$x[,1], ldpred$x[,2])

points(assigns$x[,1], assigns$x[,2])

textxy(ld1,ld2, labs = dat$Species, cex = 1.0, m =c(0,0), offset = (0.4))

#plotting

# Read in LDA scores for plotting - The LDA scores for unknowns were read in from the 'prediciton'
output above
LDAscores <- read.csv("LDAscores.csv", header=T)
LDAscores <-read.csv("LDAscores_with_sp.csv", header =T)
# Plot LDA1 vs LDA2
ggplot(LDAscores, aes(x=LD1, y = LD2, color = Species, shape = Species)) +
  geom_mark_hull(concavity = 15, expand = 0, radius = 0, aes(fill = Species, filter = Species !=
'versicolor')) +
  geom_point(size = 3) +
  # Set the Y and X axis limits
  ylim(-4, 6) +
  xlim(-6, 8) +
  # Add axes labels
  xlab("LD1 (84.32%)") +
  ylab("LD2 (14.87%)") +
  # Set the shapes manually
  scale_shape_manual(values=c(15, 3, 17, 18)) +
  # Set the colours manually
  scale_color_manual(values=c('green', 'black', 'red', 'blue')) +
  # Add text labels to point'
  geom_text(aes(label=IDs),hjust=0, vjust=0) +
  # Set the theme to classic
  theme_classic()

ggplot(LDAscores, aes(x=LD1, y = LD3, color = Species, shape = Species)) +
  geom_mark_hull(concavity = 15, expand = 0, radius = 0, aes(fill = Species, filter = Species !=
'versicolor')) +
  geom_point(size = 3) +
  # Set the Y and X axis limits
  ylim(-3, 5) +
  xlim(-5, 7) +
  # Add axes labels
```

```
  xlab("LD1 (84.32%)") +
  ylab("LD3 (0.81%)") +
  # Set the shapes manually
  scale_shape_manual(values=c(15, 3, 17, 18, 21)) +
  # Set the colours manually
  scale_color_manual(values=c('green', 'black', 'red', 'blue', 'orange')) +
  # Add text labels to point'
  geom_text(aes(label=IDs),hjust=0, vjust=0) +
  # Set the theme to classic
  theme_classic()
```

Body mass ANOVAs

```
library(dplyr)
library(ggpubr)
library(viridis)

#Alberdi ANOVAs

data<-read.csv("Alberdi_BM_anova_sp.csv", header = TRUE)
data

group_by(data, Species) %>%
  summarise(
    count = n(),
    mean = mean(Average, na.rm = TRUE),
    sd = sd(Average, na.rm = TRUE)
  )

ggboxplot(data, x = "Species", y = "Average",
          fill = "Species",
          ylab = "Alberdi Average Body Mass (kg)", xlab = "Species") +
  scale_color_viridis(discrete = TRUE, option = "D") + scale_fill_viridis(discrete = TRUE)

res.aov<-aov(Average ~ Species, data = data)
res.aov
summary.aov(res.aov)

TukeyHSD(res.aov)

#Scott ANOVAs

data2<-read.csv("Scott_BM_anovasp.csv", header = TRUE)
data2

group_by(data2, Species) %>%
  summarise(
```

```
    count = n(),
    mean = mean(Average, na.rm = TRUE),
    sd = sd(Average, na.rm = TRUE)
  )

ggboxplot(data2, x = "Species", y = "Average",
          fill ="Species",
          ylab = "Scott Average Body Mass (kg)", xlab = "Species") +
  scale_color_viridis(discrete = TRUE, option = "D") + scale_fill_viridis(discrete = TRUE)

res.aov2<-aov(Average ~ Species, data = data2)
res.aov2
summary.aov(res.aov2)

TukeyHSD(res.aov2)
```
